# Up-Regulation of the Cardiac Lipid Metabolism at the Onset of Heart Failure

**DOI:** 10.2174/187152511797037583

**Published:** 2011-07

**Authors:** Said AbdAlla, Xuebin Fu, Sherif S Elzahwy, Kristin Klaetschke, Thomas Streichert, Ursula Quitterer

**Affiliations:** 1 Molecular Pharmacology Unit, Swiss Federal Institute of Technology and University of Zurich, Zurich, Switzerland; 2 Cardiology Department, Ain Shams University Hospitals, Cairo, Egypt; 3 Department of Clinical Chemistry/Central Laboratories, University Medical Centre Hamburg-Eppendorf, Hamburg, Germany

**Keywords:** Aortic constriction, atherosclerosis, heart failure, lipid metabolism, microarray gene expression analysis, pressure overload, ranolazine.

## Abstract

Chronic pressure overload and atherosclerosis are primary etiologic factors for cardiac hypertrophy and failure. However, mechanisms underlying the transition from hypertrophy to heart failure are incompletely understood. We analyzed the development of heart failure in mice with chronic pressure overload induced by aortic constriction and compared the results with aged apolipoprotein E-deficient mice suffering from advanced atherosclerosis. We combined cardiac function analysis by echocardiography and invasive hemodynamics with a comprehensive microarray gene expression study (GSE25765-8). The microarray data showed that the onset of heart failure induced by pressure overload or advanced atherosclerosis was accompanied by a strong up-regulation of key lipid metabolizing enzymes involved in fat synthesis, storage and oxidation. Cardiac lipid overload may be involved in the progression of heart failure by enhancing cardiomyocyte death. Up-regulation of the cardiac lipid metabolism was related to oxygen and ATP depletion of failing hearts because anti-ischemic treatment with ranolazine normalized the cardiac lipid metabolism and improved cardiac function. Vice versa, inhibition of cellular respiration and ATP generation by mild thiol-blocking with cystamine triggered the cardiac lipid metabolism and caused signs of heart failure. Cardiac tissue specimens of patients with heart failure also showed high protein levels of key fat metabolizing enzymes as well as lipid accumulation. Taken together, our data strongly indicate that up-regulation of the cardiac lipid metabolism and myocardial lipid overload are underlying the development of heart failure.

## INTRODUCTION

Cardiovascular disease is still the leading cause of death. Current research focuses on pathomechanisms and risk factors. Hypertension and stable atherosclerosis are major "silent" risk factors for the development cardiac disease and cardiac death. Among several pathological events, cardiac hypertrophy develops as a major consequence of persistent pressure and volume overload. Molecular and cellular changes in hypertrophied hearts that initially mediate enhanced function are considered key events in the deterioration of heart function and the development of heart failure. Likewise, hypertrophy of the left ventricle is an independent predictor of cardiovascular dysfunction, mortality and sudden death, and in many pathological conditions, precedes congestive heart failure [1]. 

Abnormal myocardial metabolism is a key feature accompanying hypertrophy-induced pathomechanisms culminating finally in heart failure [2]. Dysregulation of cardiac energy generation may account for the myocardial energy depletion, which is a major characteristic of cardiac hypertrophy and heart failure [3, 4]. The cardiac energy metabolism relying principally (up to 70 %) on free fatty acids (FFA) for energy generation is considered one factor underlying the development of energy deficiency in heart failure because fatty acid oxidation requires about 11 % more oxygen for a given quantity of ATP synthesis than do carbohydrates. Moreover, the full impact of exaggerated oxygen consumption due to cardiac FFA oxidation may be much greater *in vivo* as a switch from maximal glucose to maximal FFA utilization induces an increase in oxygen requirement by 50 to 60 % in animal models [5]. 

In the course of heart failure, substrate utilization of free fatty acids and glucose declines, whereas plasma levels of free fatty acids, glucose and insulin are commonly increased [2, 6]. In addition to disturbed substrate utilization, the second step of cardiac energy generation, i.e. oxidative phosphorylation by cardiac mitochondria, is also impaired in heart failure [2]. Inefficient oxidative phosphorylation by the failing heart could be in part due to the increased expression of uncoupling proteins (UCP), which cause mitochondria to produce heat rather than ATP [7, 8]. Cardiac UCP expression may be directly related to the increased plasma levels of free fatty acids in heart failure because infusion of free fatty acids triggers the expression and activation of UCP under experimental conditions [8, 9].

In view of the increasing knowledge of the metabolic dysfunction in the course of heart failure, the concept arises that metabolic intervention could provide an adjuvant therapy for heart failure [6, 10]. However, currently it is not quite clear whether a shift from fatty acid towards glucose utilization should be considered beneficial or detrimental, because inhibition of mitochondrial fatty acid oxidation may enhance the accumulation of triglycerides and potentially toxic fatty acid intermediates thereby aggravating lipotoxicity [6]. 

To further understand the complex events marking the transition from cardiac hypertrophy to heart failure we performed whole genome microarray gene expression profiling of different experimental models of cardiac failure triggered by pressure overload and advanced atherosclerosis. Our study provides strong evidence that the onset of heart failure is characterized by the up-regulation of the cardiac lipid metabolism and toxic lipid overload.

## MATERIALS AND METHODS

### Chronic Pressure Overload-Induced Cardiac Hypertrophy and Heart Failure

Abdominal aortic constriction (AAC) was used to trigger pressure overload-induced cardiac hypertrophy and heart failure. All mice were fed a standard rodent chow containing 4.5 % fat. Aortic constriction of the abdominal aorta was performed in anesthetized 4 month-old male C57BL/6J (B6) mice or apolipoprotein E-deficient (ApoE^-/-^) mice, respectively, as described [11]. Briefly, the abdominal aorta was constricted between the celiac and superior mesenteric artery by tying a 7-0 silk suture ligature against a blunt 28-gauge needle. Age-matched control mice underwent identical surgical procedure except for ligation of the aorta (sham-operated mice). Cardiac parameters of mice were monitored regularly by echocardiography at 1, 2, 4 and 6 months after aortic constriction. A group of B6 mice with signs of heart failure (ejection fraction <35 %) were treated with ranolazine (200 mg/kg/d) initiated 4 months after aortic constriction.

At the end of the observation period, we determined cardiac function of all groups of mice: (i) B6 mice with 1 month of AAC, (ii) treated/untreated B6 mice with 6 months of AAC, and (iii) ApoE^-/-^ mice with 2 months of AAC. Age-matched, sham-operated ApoE^-/-^ and B6 mice served as controls. Mice were anesthetized, cardiac function parameters were determined by echocardiography and invasive hemodynamics followed by intracardial perfusion with sterile PBS. Hearts were isolated, and heart weight was determined. Hearts were immediately frozen in liquid nitrogen, or processed for further use.

### Experimental Model of Advanced Atherosclerosis-Induced Heart Failure

As a model of atherosclerosis-induced heart failure we used 18 month-old ApoE^-/-^ mice, which were on a C57BL/6J background, and C57BL/6J (B6) control mice. Until 18 months of age, ApoE^-/-^ mice were kept on a 12 h light/12 h dark regime, had free access to food and water, and were fed a rodent chow containing 7 % fat and 0.15 % cholesterol (AIN-93-based diet). To assess the effect of anti-ischemic treatment, a group of 16 month-old ApoE^-/-^ mice with signs of heart failure (ejection fraction <35 %) were treated with ranolazine (200 mg/kg/d) for 2 months. At an age of 18 months, all mice were anesthetized, and cardiac function parameters were determined by echocardiography and invasive hemodynamics.

In a separate experiment we assessed the effect of inhibition of cellular respiration. To this end a group of 16 week-old ApoE^-/-^ mice were treated for 4-6 weeks with the mild thiol-blocking agent cystamine (300 mg/kg/d). After 4-6 weeks of treatment, heart function was measured by echocardiography. At the end of the observation period, all mice were anesthetized, and after intracardial perfusion with sterile PBS, hearts were rapidly isolated, and processed for further use.

All animal experiments were performed in accordance with the NIH guidelines and reviewed and approved by the local committees on animal care and use (University of Zurich and Hamburg). 

### Echocardiography and Invasive Hemodynamics

Transthoracic echocardiography was performed on anesthetized mice with a Vivid 7 echocardiograph equipment (GE Healthcare) and a 12 MHz linear array transducer. Left ventricular dimensions, and fractional shortening were obtained in the parasternal long axis view using M-mode imaging according to the recommendations of the American Society of Cardiology [12]. Ejection fraction (EF) was calculated using the formula of Teichholz. Recordings were interpreted off-line using EchoPac Pc 3.0 software (GE)

At the end of the observation period invasive hemodynamic measurements were performed in anesthetized mice. A 1.4 French Millar catheter (Millar Instruments) was inserted into the right carotid artery and advanced into the ascending aorta and the left ventricle under continuous monitoring of the pressure waveform. Arterial and left ventricular pressures were recorded and analyzed with a PowerLab system (ADI Instruments).

### Microarray Gene Expression Analysis

Whole genome microarray gene expression analysis of cardiac tissue was performed essentially as described previously [13]. Briefly, total RNA of heart tissue was isolated with the RNeasy Midi kit (Qiagen). Procedures for cDNA synthesis, labeling and hybridization were carried out according to the protocol of the manufacturer (Affymetrix). For hybridization, 15 µg of fragmented cRNA were incubated with the chip (Affymetrix GeneChip Mouse genome MG430 2.0 Array) in 200 µl of hybridization solution in a Hybridization Oven 640 (Affymetrix) at 45 °C for 16 h. GeneChips were washed and stained using the Affymetrix Fluidics Station 450 according to the GeneChip Expression Analysis Technical Manual (Rev. 5). Microarrays were scanned with the Affymetrix GeneChip Scanner 7G, and the signals were processed with a target value of 300 using GCOS (v. 1.4, Affymetrix). All microarray gene expression data are available at NCBI GEO database accession no. GSE25765-8 (GSE25765, GSE25766, GSE25767, GSE25768).

### Antibodies

The following antibodies were used for immunohistochemistry, immunofluorescence, and immunoblotting: rabbit anti-FASN antibodies, which were raised against an antigen encompassing amino acids 2205-2504 of human fatty acid synthase (FASN); rabbit anti-Scd1 antibodies, which were raised against the amino terminal region of mouse stearoyl-Coenzyme A desaturase 1 (Scd1); rabbit anti-SCD1 antibodies, which were raised against an antigen encompassing amino acids 21-51 of human SCD1; mouse monoclonal antibody raised against α-sarcomeric actin (A2172 clone 5C5, Sigma); rabbit anti-NPPA antibodies, which were raised against an epitope encompassing amino acids 1-153 of human natriuretic peptide type A (NPPA); rabbit anti-Ucp1/UCP1 antibodies, which were raised against antigens encompassing amino acids 288-302 of mouse/human uncoupling protein 1 (Ucp1/UCP1); rabbit anti-resistin antibodies, which were raised against recombinant human resistin (RETN); rabbit anti-GAPDH antibodies, which were raised against amino acids 1-335 of human glyceraldehyde-3-phosphate dehydrogenase (GAPDH). Immunoblotting and immunohistochemistry were routinely used to determine and confirm cross-reactivity of the antibodies with the respective mouse and human proteins.

### Functional Assays

Total cardiac lipids were extracted from pulverized frozen cardiac tissue by the method of Folch *et al*. [14]. Myocardial triglyceride content was quantified using an enzymatic spectrophotometric kit according to the manufacturer`s instructions (Sigma). Lipid synthesis was determined with cardiomyocytes incubated with 1,2-[^14^C]-acetate (57 mCi/mmol; 3 µCi/dish) added to the culture medium. After 4 h of incubation at 37 °C, cells were washed with PBS, trypsinized, collected by centrifugation and resuspended in 0.8 ml PBS. Lipids were extracted as described above, and incorporation of 1,2-[^14^C]-acetate into lipids was quantified by scintillation counting. Neonatal rat cardiomycytes were isolated and transfected as described [15]. DNA strand breaks were determined by the terminal deoxynucleotidyl transferase-mediated dUTP nick-end labeling (TUNEL) technique (Roche Applied Science) essentially as detailed previously [16]. Caspase-3 activity was measured using a commercially available caspase-3 assay kit (Clontech). Measurement of [ATP] of cardiac tissue extracts was performed with a luciferase enzyme kit according to the manufacturer`s instructions (Invitrogen).

### Immunohistology

For immunohistology, paraffin sections or cryosections of mouse or human heart specimens were used. Immunohistological detection of Fasn (FASN), Ucp1 (UCP1), and Scd1 (SCD1) was performed with affinity-purified, polyclonal antibodies similarly as described [13, 17]. Oil red O staining was performed on frozen heart sections and on isolated aortas as described [13]. All sections were imaged with a Leica DMI6000 microscope equipped with a DFC420 camera.

### Immunofluorescence

Immunofluorescence localization studies were performed with cryosections (10 µm) of post-fixed and frozen hearts obtained from 10 month-old mice with 6 months of AAC. For co-localization of Fasn and α-sarcomeric actin, affinity-purified rabbit anti-Fasn antibodies and mouse anti-α-sarcomeric actin antibodies were applied (dilution 1:4000), followed by secondary antibodies or F(ab)_2_ fragments of the antibodies labeled with Alexa Fluor 546 and Alexa Fluor 488, respectively (dilution 1:5000), and counterstaining with DAPI. Sections were imaged with a Leica (TCS) confocal laser microscope.

### Immunoblotting

Immunoblot detection of proteins was performed with affinity-purified antibodies or F(ab)_2_ fragments of the respective antibodies [13]. Bound antibody was visualized with F(ab)_2_ fragments of enzyme-coupled secondary antibodies, or by enzyme-coupled protein A, as applicable, followed by enhanced chemiluminescence detection.

### Patients

Small myocardial biopsy specimens were obtained from nine patients (age 59±13 years) undergoing mitral valve replacement. Five patients had congestive heart failure (NYHA class III-IV). Informed consent was obtained from all participants. The study was conducted in conformity with the principles of the declaration of Helsinki, with approval of the protocol by the ethical committee of Ain Shams University.

### Statistical Analyses

Results are presented as mean ± S. D. unless specified otherwise. Unpaired or paired Student t tests were used to compare results between two groups. Statistical significance was set at a *p* value of < 0.05, unless indicated differently.

## RESULTS

### Chronic Pressure Overload Induces Signs of Heart Failure in B6 Mice

**Fig. (1):**
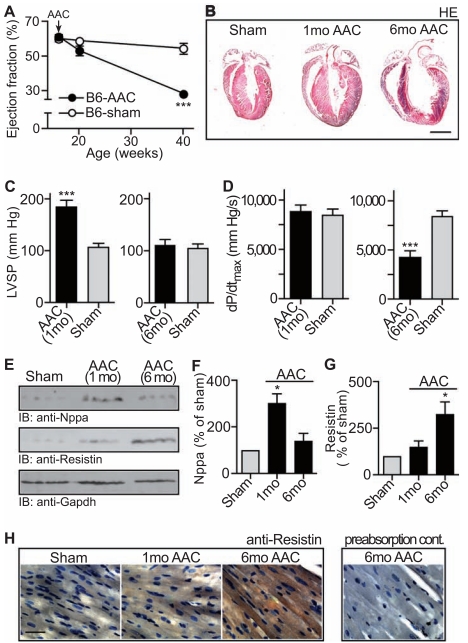
Chronic pressure overload induces signs of heart failure in B6 mice. **A**. The ejection fraction of 5 month-old and 10 month-old B6 mice with 1 month and 6 months of abdominal aortic constriction (B6-AAC), respectively, was determined by echocardiography relative to age-matched sham-operated controls (B6-sham). Abdominal aortic constriction (AAC) was performed with 4 month-old (i.e. 16 week-old) mice. Age of mice is given in weeks. Data represent mean ± S.D., n=4 mice/group (***, *p*<0.0003). **B**. Representative hematoxylin-eosin (HE)-stained heart sections from a 5 month-old (middle) and 10 month-old (right) B6 mouse isolated after 1 month (1mo AAC) and 6 months (6mo AAC) of AAC, respectively. The sham-operated control (left, Sham) is a 10 month-old mouse (bar: 2 mm). **C**, **D**. Cardiac function parameters were determined by invasive hemodynamics of 5 month-old (left) and 10 month-old (right) B6 mice. The peak left ventricular systolic pressure (LVSP; C), and the maximum rate of change of left ventricular pressure (dP/dt_max;_ **D**) were determined in B6 mice after 1 month (AAC 1mo) and 6 months (AAC 6mo) of AAC, respectively, relative to age-matched, sham-operated controls (Sham). Data represent mean ± S.D., n=4 mice/group (***, *p*<0.0004). **E**-**G**. Panel **E** shows the immunoblot detection of natriuretic peptide type A (IB: anti-Nppa), resistin (IB: anti-Resistin) and Gapdh (IB: anti-Gapdh) in heart tissue isolated from 5 month-old B6 mice after 1 month of AAC (1mo AAC) and from 10 month-old B6 mice after 6 months of AAC (6mo AAC), respectively. The sham controls were obtained from 10 month-old mice (Sham). Quantification of Nppa protein levels by densitometric immunoblot scanning is shown in **F**, and panel **G** shows the immunoblot quantification of resistin protein levels. Data represent mean ± S. D., n=4 mice/group (*, *p*<0.01; 6 months of AAC vs. 1 month of AAC). **H**. Immunohistology detection of resistin with anti-resistin antibodies (anti-Resistin) on frozen heart sections of sham-operated B6 mice (Sham, age 10 months), 5 month-old B6 mice with 1 month of AAC (1mo AAC) and 10 month-old B6 mice with 6 months of AAC (6mo AAC). Nuclei were stained with hematoxylin (bar: 40 µm). As specificity control, pre-absorption of the antibodies with the antigen used for immunization (preabsorption cont.) was performed. Histology data are representative of at least 3 different mice per group (**B**, **H**).

Chronically elevated blood pressure is a major causative factor for the development of cardiac hypertrophy culminating finally in heart failure [18]. To analyze mechanisms underlying the transition from cardiac hypertrophy to heart failure we used an experimental model of chronic pressure overload induced by abdominal aortic constriction (AAC). In agreement with previous studies [19], echocardiography showed that 1 month of aortic constriction did not significantly impair the cardiac function of B6 mice compared to sham-operated controls (Fig. **1A**). In contrast, after 6 months of chronic pressure overload, B6 mice showed signs of heart failure as evidenced by the strongly reduced ejection fraction of 28.8 ± 3.3 % (Fig. **1A**, *p<0.0003 vs. sham-operated, age-matched B6 mice).

As demonstrated by others [19], 1 month of aortic constriction induced cardiac hypertrophy in B6 mice (Fig. **1B**, middle vs. left panel). However, after 6 months of pressure overload, histological analysis demonstrated that hearts of B6 mice with signs of heart failure were characterized by cardiac hypertrophy with dilation (Fig. **1B**, right vs. middle and left panel).

In addition to dilated cardiac hypertrophy, invasive hemodynamics demonstrated the severe impairment of heart function after 6 months of AAC. While the peak left ventricular systolic pressure was not altered in mice with severe cardiac dilation (Fig. **1C**, right vs. left panel), there was a significant decrease in the maximum rate of change of left ventricular pressure, dP/dt_max, _after 6 months of AAC compared to sham-operated controls (Fig. **1D**, right vs. left panel). For comparison, mice with 1 month of AAC showed a significantly increased peak left ventricular systolic pressure whereas the maximum rate of change of left ventricular pressure was maintained relative to sham-operated control mice (Fig. **1C,D**, left panels).

As an additional control, 1 month of AAC induced a significant rise in cardiac protein levels of the hypertrophic marker, natriuretic peptide type A (Nppa). In contrast, Nppa levels returned to baseline after 6 months of AAC during the transition to heart failure (Fig. **1E**, upper panel, and Fig. **1F**; and ref. [20]).

To further analyze the development of signs of heart failure induced by 6 months of AAC, cardiac resistin was determined because resistin may serve as an indicator of heart failure in patients [21]. Quantitative immunoblotting revealed that cardiac resistin protein levels were significantly higher in mice with signs of heart failure whereas resistin was not different from control mice after 1 month of AAC (Fig. **1E**, middle panel, and Fig. **1G**). Immunohistology confirmed the increased resistin protein levels in the hypertrophic myocardium with dilation after 6 months of aortic constriction while resistin was not significantly increased in the hypertrophic heart after 1 month of AAC compared to sham-operated controls (Fig. **1H**, right vs. middle and left panel). Together the data indicate that 6 months of AAC induced signs of heart failure with dilated cardiac hypertrophy in B6 mice.

### Pressure Overload Accelerates the Development of Heart Failure in Atherosclerosis-Prone ApoE^-/-^ Mice

**Fig. (2):**
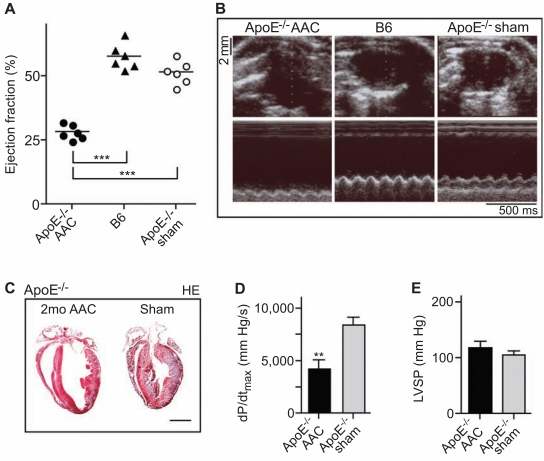
Pressure overload accelerates the development of heart failure in ApoE^-/-^ mice. **A**. Ejection fraction of 6 month-old ApoE^-/-^ mice after 2 months of abdominal aortic constriction (ApoE^-/-^ AAC) relative to age-matched B6 mice (B6) and sham-operated ApoE^-/-^ mice (ApoE- /- sham) was determined by echocardiography (***, *p*<0.0001, n=6). **B**. Representative echocardiograms of a 6 month-old ApoE^-/-^ mouse after 2 months of AAC (ApoE^-/-^ AAC; left panels), an age-matched B6 mouse (B6; middle panels), and a 6 month-old, sham-operated ApoE^-/-^ mouse (ApoE^-/-^ sham; right panels) in B-mode (upper panels) and M-mode (lower panels). **C**. Representative hematoxylin-eosin (HE)-stained heart sections of a 6 month-old ApoE^-/-^ mouse with 2 months of AAC (2mo AAC; left panel) and a 6 month-old sham-operated ApoE^-/-^ control mouse (Sham; right panel). Histology data are representative of at least 3 different mice per group (bar: 2 mm). **D**, **E**. Cardiac function parameters were determined by invasive hemodynamics of 6 month-old ApoE^-/-^ mice after 2 months of AAC (ApoE^-/-^ AAC) and of shamoperated ApoE^-/-^ controls (ApoE^-/-^ sham). The maximum rate of change of left ventricular pressure (dP/dt_max_; **D**), and the peak left ventricular systolic pressure (LVSP; **E**) are shown. Data represent mean ± S.D., n=4 mice/group (**, *p*<0.0004).

In addition to pressure overload, atherosclerosis is another major etiologic factor for the development of heart failure [22, 23]. To analyze the impact of atherosclerosis on the development of heart failure induced by chronic pressure overload, aortic constriction was performed in atherosclerosis-prone ApoE^-/-^ mice. In contrast to B6 mice (cf. Fig. **1**), 2 months of pressure overload were sufficient to induce signs of heart failure in ApoE^-/-^ mice (Fig. **2A**). After 2 months of aortic constriction, the ejection fraction of ApoE^-/-^ mice was reduced to 28.1 ± 2.9 % (*p<0.0001 vs. sham-operated B6 or ApoE^-/-^ mice). As a control, the cardiac function was not significantly different between sham-operated 6 month-old B6 and ApoE^-/-^ mice (Fig. **2A, B**).

Concomitantly to signs of heart failure, echocardiography showed left ventricular dilation in ApoE^-/-^ mice after two months of aortic constriction (Fig. **2B**). The left ventricular end diastolic diameter was 7.2 ± 0.2 mm, 5.9 ± 0.3 mm and 6.1 ± 0.3 mm of aortic-constricted ApoE^-/-^, B6 and sham-operated ApoE^-/-^ mice, respectively (n=6 mice per group, *p<0.0002 between aortic-constricted ApoE^-/-^, and B6 or sham-operated ApoE^-/-^ mice).

Histological analysis of hematoxylin- and eosin-stained heart sections confirmed that two months of pressure overload had induced cardiac hypertrophy with dilation in ApoE^-/-^ mice compared to sham-operated control mice (Fig. **2C**, left vs. right panel).

In addition to the strongly reduced ejection fraction there was a significant reduction in the maximum rate of change of left ventricular pressure (dP/dt_max_) upon two months of AAC relative to sham-operated ApoE^-/-^ mice, as determined by invasive hemodynamics (Fig. **2D**). Moreover, the peak left ventricular systolic pressure of aortic-constricted ApoE^-/-^ mice with dilated cardiac hypertrophy was comparable to the left ventricular systolic pressure of sham-operated controls (Fig. **2E**). Together these data show that two months of pressure overload had induced signs of heart failure in ApoE^-/-^ mice.

### Aged ApoE^-/-^ Mice Develop Signs of Heart Failure and Dilated Cardiac Hypertrophy

**Fig. (3):**
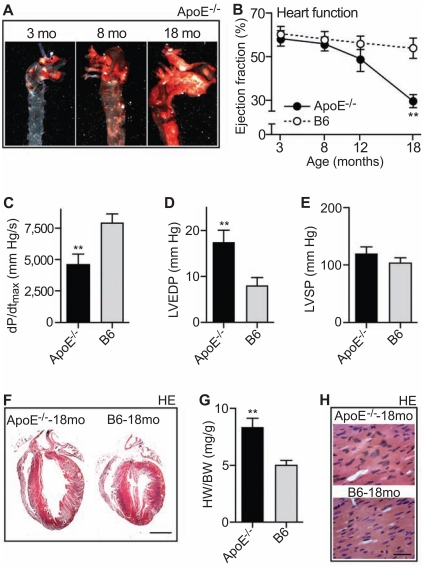
Aged ApoE^-/-^ mice develop signs of heart failure and dilated cardiac hypertrophy. **A**. Detection of atherosclerotic plaques in the aorta of ApoE^-/-^ mice (ApoE^-/-^) by oil red O staining. Aortas were isolated at 3, 8 and 18 months (mo) of age. **B**. The ejection fraction was determined by echocardiography of 3, 8, 12 and 18 month-old ApoE^-/-^ mice relative to B6 mice (± S. D., n=4; **, *p*<0.0002; ApoE^-/-^ vs. age-matched B6 mice). **C**-**E**. Cardiac function parameters of 18 month-old ApoE^-/-^ mice were determined by invasive hemodynamics. Panel **C** shows the maximum rate of change of left ventricular pressure (dP/dt_max_). Panels **D** and **E** present the left ventricular end-diastolic pressure (LVEDP; **D**) and the peak left ventricular systolic pressure (LVSP; **E**) of 18 month-old ApoE^-/-^ mice (ApoE^-/-^) relative to age-matched B6 control mice (± S. D.; n=4; **, *p*<0.002). **F**. Hematoxylin-eosin (HE) stained heart sections of an 18 month-old ApoE^-/-^ (left panel) and an age-matched control B6 mouse (right panel); bar: 2 mm. **G**. Increased heart weight to body weight ratio (HW/BW) of 18 month-old (18mo) ApoE^-/-^ mice relative to age-matched B6 mice (±S.D., n=4; **, *p*<0.0002). **H**. Hematoxylin eosin (HE)-stained heart sections of an 18 month-old ApoE^-/-^ mouse and an age-matched control mouse (bar: 40 µm). Histology data are representative of at least 3 different mice per group (**A**, **F**, **H**).

Chronic atherosclerosis is a primary etiologic risk factor for the development of heart failure in patients [22, 23]. However, the relationship between atherosclerosis and the development of heart failure in an animal model of atherosclerosis is not established. To analyze the impact of atherosclerosis on heart function, we used aged ApoE^-/-^ mice. With increasing age, ApoE^-/-^ mice developed a high atherosclerotic plaque load in the aorta (Fig. **3A**). At an age of 18 months, the transverse aorta of ApoE^-/-^ mice was almost completely covered with atherosclerotic plaques (Fig. **3A**, right vs. middle and left panel). The aortic surface area covered with lesions was 8.0 ± 2.4 %, 24.2 ± 4.9 % and 88.0 ± 4.2 % of 3, 8 and 18 month-old ApoE^-/-^ mice, respectively, as determined by quantitative image analysis of oil red O stained aortas (n = 5 mice/group).

We asked whether the development of atherosclerotic plaques in the aorta affected the cardiac function of ApoE^-/-^ mice. Echocardiography showed that the increase in atherosclerotic plaque area was accompanied by a strongly reduced ejection fraction (Fig. **3B**). At an age of 18 months, the ejection fraction of ApoE^-/-^ mice was 29.7 ± 2.9 % whereas age-matched C57BL/6J (B6) control mice had an ejection fraction of 54.0 ± 4.7 % (Fig. **3B**). Invasive hemodynamics further confirmed the impaired cardiac function of 18 month-old ApoE^-/-^ mice. The maximum rate of change of left ventricular pressure (dP/dt_max_) was significantly reduced of ApoE^-/-^ mice relative to age-matched B6 mice (Fig. **3C**). Concomitantly, there was a significant increase in the left ventricular end-diastolic pressure (Fig. **3D**) while the peak left ventricular systolic pressure was not significantly different between 18 month-old ApoE^-/-^ and B6 control mice (Fig. **3E**).

In addition to the impaired cardiac function, hearts of 18 month-old ApoE^-/-^ mice with high atherosclerotic plaque load displayed signs of dilated cardiac hypertrophy as determined by histological analysis of hematoxylin/eosin (HE)-stained sections (Fig. **3F**). The heart weight to body weight 

ratio was also significantly increased (Fig. **3G**), and cardiomyocyte hypertrophy was visible by an increased cell size and nuclear size of hypertrophied cardiomyocytes compared to B6 controls (Fig. **3H**). Altogether, the data reveal a strongly impaired heart function and signs of heart failure in aged ApoE^-/-^ mice with advanced atherosclerosis.

### Microarray Gene Expression Profiling Demonstrates Up-Regulation of the Cardiac Lipid Metabolism at the Onset of Heart Failure

**Fig. (4):**
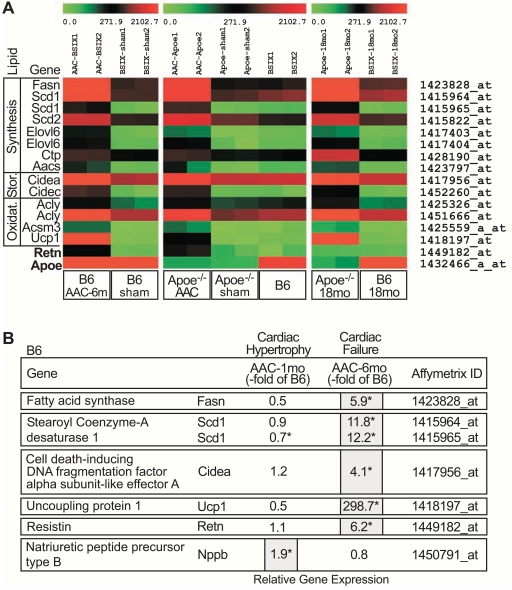
Up-regulation of the cardiac lipid metabolism at the onset of heart failure. **A**. Microarray gene expression analyses of heart tissue from three different models of heart failure were compared to respective controls: (i) 10 month-old B6 mice with 6 months of AAC (B6 AAC-6m) relative to 10 month-old, sham-operated B6 controls (B6 sham), (ii) 6 month-old ApoE^-/-^ mice with 2 months of AAC (Apoe^-/-^ AAC) relative to age-matched, sham-operated ApoE^-/-^ controls (Apoe^-/-^ sham), and (iii) 18 month-old ApoE^-/-^ mice (Apoe^-/-^ 18 mo) relative to 18 month-old B6 controls (B6 18mo). As an additional control for ApoE^-/-^ AAC model, age-matched B6 mice (B6) are shown. Normalized signal intensity values of differentially expressed probe sets detecting fat metabolism genes are presented as heat map centered to the median value. Probe sets, which showed (i) a significantly different signal intensity value relative to the respective controls (*, *p*<0.01), (ii) a more than 2-fold up-regulation relative to the respective controls, and (iii) involvement in fat metabolism are listed. Probe sets detecting genes involved in lipid synthesis (Synthesis), storage (Stor.) and oxidation (Oxidat.) are shown. As controls, the probe sets detecting resistin (Retn) and ApoE (Apoe) were included. **B**. Microarray gene expression analysis data showed up-regulation key fat metabolism genes in failing cardiac tissue isolated from 10 month-old B6 mice after 6 months of AAC (Cardiac Failure; AAC-6mo) whereas the expression of fat metabolism genes was normal in 5 month-old B6 mice after 1 month of AAC (Cardiac Hypertrophy; AAC-1mo) inducing cardiac hypertrophy without failure (cf. Fig. **1**). Gene expression is presented as fold change relative to the respective age-matched, sham-operated B6 control (-fold of B6; *, *p*<0.01).

To identify genes specifically up-regulated at the onset of heart failure we performed a whole genome microarray gene expression study with cardiac tissue from the three experimental models of heart failure. After data normalization, microarray data were subjected to stringent data filtering according to the following criteria: (i) significant difference from the respective control group (*p<0.01), (ii) more than two-fold up-regulation relative to control values, and (iii) involvement in lipid metabolism according to the literature. We focused on proteins involved in lipid synthesis and metabolism because metabolic dysfunction is a key feature of the failing heart, and serum levels of free fatty acids are increased in the course of heart failure in patients [2, 6]. Moreover, excessive triglyceride and lipid accumulation is a characteristic pathologic feature of failing and ischemic heart tissue [24, 25]. With those stringent criteria, 11 fat metabolism genes were identified, which are essentially involved in lipid synthesis, storage and oxidation (Fig. **4A**). Notably, those 11 genes were similarly up-regulated in the three different models of heart failure where heart failure was induced by pressure overload or advanced atherosclerosis (Fig. **4A**, left, middle, right panels). As a control, the cardiac lipid metabolism gene cluster was not induced by hypercholesterolemia because expression of the lipid metabolism genes was not different between young ApoE^-/-^ mice (age 24 weeks, without heart failure signs) and age-matched B6 mice (Fig. **4A**, middle panel).

Specifically, the microarray approach showed up-regulation of key enzymes of fatty acid synthesis, i.e. Fasn (fatty acid synthase), Scd1, Scd2 (stearoyl-Coenzyme A desaturase 1 and 2), and Elovl6 (long chain fatty acid elongase 6). Two up-regulated genes are known to increase the supply of acetyl CoA/acetoacetyl CoA as primers of fatty acid synthesis, i.e. Ctp (citrate transport protein) and Aacs (acetoacetyl-Coenzyme A synthase). And Cidea and Cidec (cell death-inducing DNA fragmentation factor, alpha subunit-like effector A and C) stimulate the storage of lipids in lipid droplets. In addition to enzymes stimulating fatty acid synthesis and storage, another group of up-regulated genes is involved in mitochondrial fatty acid oxidation: Acly (ATP citrate lyase), Acsm3 (acyl-CoA synthetase medium chain family member 3) and Ucp1 (uncoupling protein 1). 

The up-regulation of the cardiac lipid metabolism was related to the onset of heart failure signs, because hypertrophic heart tissue isolated from B6 mice after 1 month of AAC without signs of heart failure (cf. Fig. **1**) did not show a significant change in the expression of lipid metabolism genes, although the hypertrophic marker, natriuretic peptide type B (Nppb), was increased (Fig. **4B**). As controls, the heart failure-related protein, resistin (Retn), was specifically up-regulated in aortic-constricted mice with signs of heart failure (Fig. **4A**, left and middle panel; Fig. **4B**) while ApoE was only expressed in B6 mice (Fig. **4A**). Thus, the micro-array approach revealed the strong up-regulation of major fat metabolizing enzymes in hearts of mice with signs of heart failure.

### Myocardial Fatty Acid Synthase Protein in Failing Hearts

**Fig. (5):**
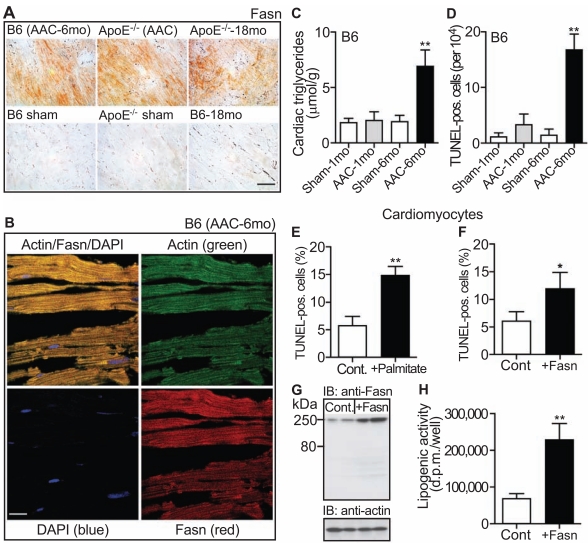
Myocardial fatty acid synthase, lipid accumulation and cell death. **A**. Immmunohistology detection of Fasn with anti-Fasn antibodies (Fasn) on frozen heart sections of three different heart failure models (upper panels), i.e. 10 month-old B6 mice with 6 months of AAC (B6 AAC-6mo), 6 month-old ApoE^-/-^ mice with 2 months of AAC (ApoE^-/-^ AAC), and 18 month-old ApoE^-/-^ mice (ApoE^-/-^-18 mo). Age-matched, 10 month-old sham-operated B6 mice (B6 sham), 6 month-old sham-operated ApoE^-/-^ mice (ApoE^-/-^ sham) and 18 month-old B6 mice (B6-18 mo) served as controls (lower panels; bar: 40 µm). **B**. Immunofluorescence co-localization of Fasn with α-sarcomeric actin on frozen heart sections of a 10 month-old B6 mouse with 6 months of AAC (B6 AAC-6mo). α-sarcomeric actin was detected with affinity-purified, mouse anti-actin antibodies followed by F(ab)_2_ fragments of Alexa Fluor 488-labeled (green) secondary antibodies (upper left/right panels). The actin-positive cardiomyocytes co-localized with Fasn, which was detected with affinity-purified, rabbit anti-Fasn antibodies followed by F(ab)_2_ fragments of Alexa Fluor 546-labeled (red) secondary antibodies (upper left/lower right panels). Cell nuclei were stained with DAPI (blue, left panels; bar: 20 µm). Immunohistology (**A**) and immunofluorescence (**B**) data are representative of at least 3 different mice/group. **C**. Cardiac triglyceride content (µmol/g wet weight) was determined with 5 month-old B6 mice after 1 month of AAC (AAC-1mo) and 10 month-old B6 mice after 6 months of AAC (AAC-6mo). Age-matched sham-operated B6 mice served as controls (Sham-1mo; Sham-6mo). Data represent mean ± S.D., n=4 mice/group (**, *p*<0.001; 6 months of AAC vs. 1 month of AAC). **D**. The number of TUNEL-positive cardiomyocytes (per 10^4^ cells) was determined by an *in situ* cell death detection (TUNEL) assay on formalin-fixed, paraffin-embedded heart sections of 5 month-old B6 mice after 1 month of AAC (AAC-1mo) and 10 month-old B6 mice after 6 months of AAC (AAC-6mo). Agematched, sham-operated B6 mice served as controls (Sham-1mo; Sham-6mo). Data represent mean ± S. D., n=4 mice/group (**, *p*<0.001; 6 months of AAC vs. 1 month of AAC). **E**, **F**. Percentage (%) of TUNEL-positive cardiomyocytes was determined (**E**) with isolated neonatal cardiomyocytes incubated with 0.5 mM palmitic acid/BSA for 18 h (+Palmitate) relative to control cells (Cont.), and (**F**) with isolated cardiomyocytes over-expressing Fasn (+Fasn) relative to mock-transfected control cardiomyocytes (Cont.). Data represent mean ± S.D., n=3 different experiments performed in triplicates each (**, *p*<0.0001 and *, *p*<0.005). **G**. Over-expression of Fasn in transfected cardiomyocytes (+Fasn) was detected in immunoblot with Fasn-specific antibodies (IB: anti-Fasn) relative to mock-transfected cells (Cont.). The lower panel is a control immunoblot detecting actin (IB: anti-actin). **H**. Transfected cardiomyocytes expressing Fasn show increased lipogenic activity relative to mock-transfected controls. Lipogenic activity was determined by incorporation of 1,2-[^14^C]-acetate into lipids and is expressed as d.p.m. per well. Data represent mean ± S. D., n=3 different experiments performed in triplicates each (**, *p*<0.0005).

**Fig. (6):**
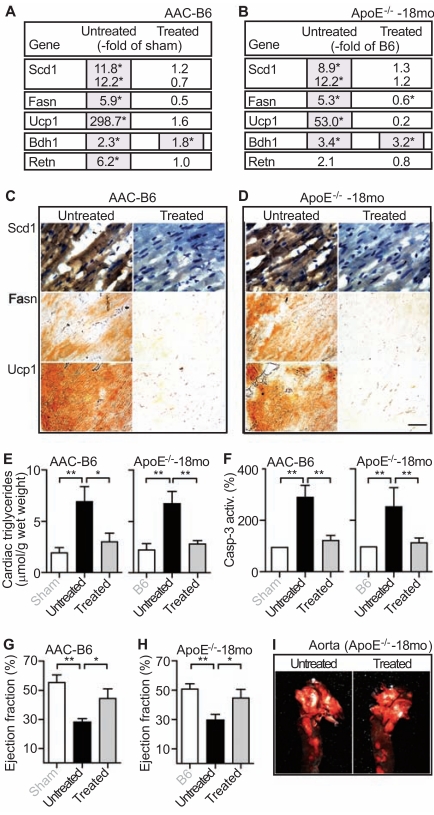
Anti-ischemic treatment with ranolazine normalized the cardiac lipid metabolism and cardiac function of mice with signs of heart failure. **A**, **B**. The effect of 2 months of ranolazine treatment (Treated) on the expression of lipid metabolism genes was determined by microarray analysis of hearts isolated from 10 month-old B6 mice with 6 months of AAC (**A**, AAC-B6; Untreated vs. Treated), and 18 month-old ApoE^-/-^ mice with advanced atherosclerosis (**B**, ApoE^-/-^-18mo; Untreated vs. Treated). Gene expression is presented as fold change relative to the respective control, i.e. untreated 10 month-old sham-operated B6 mice (**A**, -fold of sham), and untreated 18 month-old B6 mice (**B**, -fold of B6); *, *p*<0.01. **C**, **D**. Immunohistological detection of Scd1 (upper panels), Fasn (middle panels) and Ucp1 (lower panels) was performed with the respective antibodies on heart sections of untreated (Untreated; left panels) and ranolazine-treated (Treated; right panels) 10 month-old B6 mice after 6 months of AAC (**C**, AACB6), and 18 month-old ApoE^-/-^ mice (**D**, ApoE^-/-^-18mo). Histology data are representative of at least 3 different mice/group (bar: 40 µm). Nuclei were stained with hematoxylin (upper panels). **E**. Cardiac triglyceride content was determined with hearts isolated from untreated (Untreated) and ranolazine- treated (Treated) 10 month-old B6 mice after 6 months of AAC (AAC-B6; left panel), and 18 month-old ApoE^-/-^ mice (ApoE^-/-^-18mo; right panel). As controls, 10 month-old, sham-operated B6 mice (Sham, left panel), and 18 month-old B6 mice (B6, right panel) were included. Data represent mean ± S. D., n=4 mice/group (**, *p*<0.001; *, *p*<0.01). **F**. Caspase-3 activity (Casp-3 activity) as a marker of apoptotic cell death was determined with hearts isolated from untreated (Untreated) and ranolazine-treated (Treated) 10 month-old B6 mice after 6 months of AAC (AAC-B6; left panel), and 18 month-old ApoE^-/-^ mice (ApoE^-/-^-18mo; right panel). Data are expressed as percentage of the respective control (set to 100 %), i.e. 10 month-old sham-operated B6 mice (Sham, left panel), and 18 month-old B6 mice (B6, right panel), and represent mean ± S. D., n=4 mice/group (**, *p*<0.001). **G**. The ejection fraction of untreated (Untreated) and ranolazine-treated (Treated) 10 month-old B6 mice with 6 months of AAC (AAC-B6) was determined by echocardiography. Untreated, sham-operated 10 month-old B6 mice (Sham) served as controls. Data represent mean ± S. D., n=4 mice/group (**, *p*<0.0005; *, *p*<0.01). **H**. The ejection fraction (expressed in %) of untreated (Untreated) and ranolazine-treated (Treated) 18 monthold ApoE^-/-^ mice (ApoE^-/-^-18mo). Untreated 18 month-old B6 mice (B6) served as controls. Data represent mean ± S. D., n=4 mice/group (**, *p*<0.001; *, *p*<0.01). **I**. Ranolazine-treatment (Treated; right panel) for two months did not significantly affect the atherosclerotic plaque load in the aorta of 18 month-old ApoE^-/-^ mice (Aorta; ApoE^-/-^-18mo) relative to age-matched untreated ApoE^-/-^ mice (Untreated; left panel).

In agreement with the microarray data, fat metabolizing proteins identified by the microarray approach were localized in cardiac tissue of failing hearts as exemplified for the essential fat synthesizing enzyme, fatty acid synthase (Fasn) (Fig. **5A**, upper panels; and cf. Fig. **6C, D**). High protein levels of Fasn were detected by immunohistology on heart sections of three different models of heart failure (Fig. **5A**, upper panels). For comparison, protein levels of Fasn were low in cardiac tissue from control mice (Fig. **5A**, lower panels).

Co-localization studies with the cardiomyocyte-specific marker, α-sarcomeric actin, confirmed localization of up-regulated Fasn in cardiomyocytes (Fig. **5B**). 

### Increased Cardiac Triglyceride Content and Cell Death in Mice with Signs of Heart Failure

Concomitantly to increased levels of lipid synthesizing enzymes, the cardiac triglyceride content was significantly increased in B6 mice with signs of heart failure induced by 6 months of AAC (Fig. **5C**). In contrast, cardiac triglycerides after 1 month of AAC were not significantly different from sham-operated control mice (Fig. **5C**). Thus, the onset of heart failure in B6 mice was accompanied by the up-regulation of the cardiac lipid metabolism and the accumulation of cardiac lipids. 

Failing hearts of patients are characterized by increased cardiomyocyte apoptosis [26], and accumulation of lipids is considered toxic to the heart [25, 27]. To determine whether accumulation of cardiac triglycerides was accompanied by an increase in cardiomyocyte cell death, quantification of cell nuclei with DNA strand breaks as a marker of cell death was performed *in situ* by TUNEL technology. The number of TUNEL-positive cell nuclei was significantly increased in cardiac tissue of B6 mice with signs of heart failure triggered by 6 months of AAC compared to B6 mice with 1 month of AAC (Fig. **5D**). As a control, TUNEL-positive cells were almost undetectable in sham-operated control mice (Fig. **5D**). Altogether, heart failure signs in B6 mice correlated with the accumulation of cardiac triglycerides and an increase of DNA-strand breaks as a marker of cardiomyocyte cell death.

### Lipids are Toxic to Isolated Cardiomyocytes

Accumulation of lipids is considered toxic to the heart by enhancing cardiomyocyte cell death [25, 27]. In agreement with that notion, incubation of isolated cardiomyocytes for 18 h with 0.5 mM palmitic acid increased cardiomyocyte cell death as visible by more than 2.5-fold increased levels of TUNEL-positive cells with DNA strand breaks relative to control cells (Fig. **5E**).

Palmitic acid comprises more than 90 % of fatty acids synthesized by Fasn [28]. Therefore we asked whether the increased expression of Fasn in cardiomyocytes also triggered DNA strand breaks as a marker of cardiomyocyte cell death. Isolated cardiomyocytes were transfected with a plasmid encoding Fasn, and DNA strand breaks were determined. The TUNEL assay showed that cardiomyocyte death was also strongly increased by the expression of Fasn (Fig. **5F**). As a control, Fasn protein levels were increased ~3.1-fold by transfection of Fasn as determined by immunoblot with Fasn-specific antibodies (Fig. **5G**). Concomitantly, the lipogenic activity of cardiomyocytes was increased 3.3-fold (Fig. **5H**). Together the data show that fatty acid overload increased markers of cardiomyocyte death. 

### Anti-Ischemic Treatment with Ranolazine Normalized the Cardiac Lipid Metabolism at the Onset of Heart Failure

Accumulation of lipids was linked to oxygen deprivation [25], which may be triggered by the high myocardial oxygen consumption of failing hearts to meet the increased ATP demand [2, 3]. To analyze whether up-regulation of the cardiac lipid metabolism at the onset of heart failure was also related to insufficient oxygen supply, treatment was performed with an anti-ischemic drug, ranolazine [29]. The effect of ranolazine was analyzed in two experimental models of heart failure, i.e. 10 month-old B6 mice with 6 months of chronic pressure overload and 18 month-old ApoE^-/-^ mice with advanced atherosclerosis. Microarray gene expression analysis showed that ranolazine treatment for two months normalized the up-regulated fat metabolism of failing B6 hearts with 6 months of AAC-induced pressure overload as exemplified for probe sets detecting Scd1, Fasn, and Ucp1 (Fig. **6A**). Ranolazine specifically affected the fat metabolism whereas the expression of another metabolic enzyme, 3-hydroxybutyrate dehydrogenase type 1 (Bdh1) was not significantly reduced by ranolazine (Fig. **6A**).

The microarray analysis also demonstrated that ranolazine reduced the up-regulated fat metabolism of 18 month-old ApoE^-/-^ mice with heart failure (Fig. **6B**). Again, the AAC-induced up-regulation of Bdh1 was not significantly affected by ranolazine treatment (Fig. **6B**).

Immunohistological detection of fat metabolizing enzymes Scd1 and Fasn on heart sections of untreated and ranolazine-treated B6 mice with 6 months of AAC confirmed the down-regulation of key fat synthesizing enzymes by ranolazine (Fig. **6**, upper and middle panels). Similarly, immunohistology also demonstrated the ranolazine-induced normalization of Scd1 and Fasn protein levels in heart tissue of aged ApoE^-/-^ mice (Fig. **6D**, upper and middle panels). In addition to fat synthesizing enzymes, ranolazine also normalized the expression and protein levels of the fat oxidizing enzyme, Ucp1, in failing hearts of B6 mice with chronic pressure overload and ApoE^-/-^ mice with advanced atherosclerosis (Fig. **6A,B** and Fig. **6C,D**, lower panel). Thus, the anti-ischemic drug ranolazine normalized the increased lipid metabolism in two different experimental models of heart failure.

### Anti-Ischemic Treatment Reduced Cardiac Lipid Overload and Cardiomyocyte Apoptosis

Concomitantly to the normalization of fat metabolizing enzymes, ranolazine also strongly reduced the cardiac triglyceride content of the two different models of heart failure (Fig. **6E**). The reduced cardiac lipid load was accompanied by a decreased cardiomyocyte cell death as detected by the normalized activity of the apoptosis effector caspase-3 in 

heart tissue of ranolazine-treated aortic-constricted B6 and aged ApoE^-/-^ mice (Fig. **6F**). Those data show that anti-ischemic treatment normalized the cardiac lipid overload and reduced cardiomyocyte apoptosis in experimental heart failure models.

### Anti-ischemic Treatment with Ranolazine Improved the Cardiac Function of Mice with Signs of Heart Failure

Ranolazine also reduced the up-regulated heart failure marker resistin indicating a positive treatment effect on heart function (cf. Fig. **6A, B**). In agreement with that notion, echocardiography revealed a significant improvement of the impaired cardiac function of aortic-constricted B6 mice by ranolazine treatment (Fig. **6G**). The ejection fraction of untreated B6 mice after 6 months of AAC was 28.0 ± 2.6 whereas the ejection fraction of ranolazine-treated mice was 44.8 ± 6.4 % (Fig. **6G**).

Ranolazine also improved the cardiac function of aged ApoE^-/-^ mice with severe atherosclerosis. The ejection fraction was 29.5 ± 4.2 % of untreated ApoE^-/-^ mice, and 45.0 ± 5.7 % of ranolazine-treated ApoE^-/-^ mice (Fig. **6H**). As a control, two months of ranolazine treatment did not affect the development of atherosclerotic plaques in the aorta of 18 month-old ApoE^-/-^ mice (Fig. **6I**). Altogether the data show, that anti-ischemic treatment normalized the cardiac lipid metabolism at the onset of heart failure and improved the cardiac function in two different experimental models of heart failure.

### Inhibition of Cellular Respiration by Mild Thiol-Blocking with Cystamine Up-Regulated the Cardiac Lipid Metabolism and Induced Heart Failure in ApoE^-/-^ Mice

**Fig. (7):**
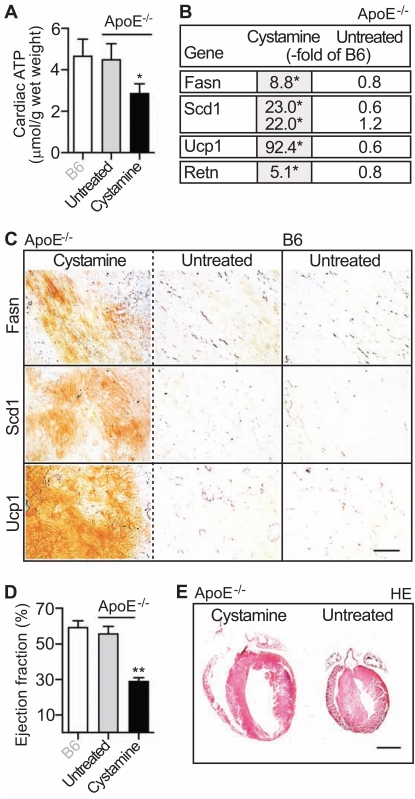
7). Inhibition of cellular respiration and ATP generation by mild thiol-blocking with cystamine up-regulated the cardiac lipid metabolism and induced heart failure in ApoE^-/-^ mice. **A**. Cardiac ATP content was determined by luminescence assay of heart tissue extracts from cystaminetreated (Cystamine; 4 weeks of treatment) ApoE^-/-^ mice (ApoE^-/-^; age 5 months) relative to age-matched untreated ApoE^-/-^ mice (Untreated) and B6 control mice (B6). Data represent mean ± S.D., n=4 mice/group (*, *p*<0.02, cystamine-treated vs. untreated). **B**. Effect of cystamine treatment (4 weeks) on the expression of lipid metabolism genes was assessed by microarray analysis with heart tissue of 5 month-old, cystamine-treated ApoE^-/-^ mice (Cystamine) relative to untreated age-matched ApoE^-/-^ mice (Untreated). Gene expression is presented as fold change relative to age-matched B6 mice (-fold of B6; *p<0.01). **C**. Immunohistological detection of Fasn (upper panels), Scd1 (middle panels) and Ucp1 (lower panels) was performed with the respective antibodies on heart sections of cystamine-treated (left panels; Cystamine) and untreated (middle panels; Untreated) 5 month-old ApoE^-/-^ mice (ApoE^-/-^). Heart sections of age-matched, untreated B6 mice (B6) are shown for comparison. Histology data are representative of at least 3 different mice/group (bar: 40 µm). **D**. The ejection fraction (expressed in %) of cystamine-treated (Cystamine) and untreated (Untreated) 5 month-old ApoE^-/-^ mice (ApoE^-/-^) was determined by echocardiography. Untreated 5 month-old B6 mice (B6) served as controls. Data represent mean ± S.D., n=4 mice/group (**, *p*<0.001, cystamine-treated vs. untreated). **E**. Hematoxylin-eosin (HE)- stained heart sections of a 5 month-old cystamine-treated ApoE^-/-^ mouse (left panel; Cystamine) and an age-matched untreated ApoE^-/-^ mouse (right panel; Untreated). Histology data are representative of at least 3 different mice/group (bar: 2 mm).

The above findings suggest a causal relationship between insufficient oxygen and ATP supply in failing hearts [2, 3] and up-regulation of the cardiac lipid metabolism. To further analyze the impact of insufficient ATP supply on cardiac lipid metabolism gene expression, we inhibited cellular respiration *in vivo* by disulfide poisoning with the mild thiol-blocking agent cystamine [30, 31]. We chose 4 month-old ApoE^-/-^ mice, which were highly vulnerable to increased oxygen and ATP demands due to pressure-overload (cf. Fig. **2**). After four weeks of cystamine treatment (300 mg/kg/d in drinking water, ref. [32]), hearts were isolated and analyzed. Mild inhibition of cellular respiration by cystamine led to a significant reduction of cardiac ATP content by 36.5 ± 10.5 % relative to untreated ApoE^-/-^ control mice (n=4, *, p<0.02; Fig. **7A**).

Next we asked whether chronic impairment of cellular respiration and ATP depletion also affected the cardiac lipid metabolism. Microarray gene expression analysis of heart tissue demonstrated a significantly increased expression of cardiac lipid metabolism genes in cystamine-treated ApoE^-/-^ mice compared to untreated controls as evidenced by the up-regulation of Fasn, Scd1 and Ucp1 (Fig. **7B**). Concomitantly, the cardiac lipid content was significantly increased (data not shown). The microarray data were confirmed by immunohistology analysis showing increased Fasn, Scd1 and Ucp1 protein levels on heart sections of cystamine-treated ApoE^-/-^ mice compared to untreated ApoE^-/-^ and B6 mice (Fig. **7C**). 

Similar to aortic-constricted mice (cf. Figs. **4, 6**), the microarray data also showed a strong up-regulation of the cardiac resistin expression in hearts of cystamine-treated ApoE^-/-^ mice (Fig. **7B**). The increased expression of the heart failure marker resistin correlated with a strongly reduced cardiac ejection fraction of 29.0 ± 2.2 % confirming the development of heart failure upon cystamine treatment (Fig. **7D**). In agreement with heart failure, the histology analysis of hematoxylin-eosin stained heart sections revealed cardiac hypertrophy with dilation after cystamine-treatment (Fig. **7E**). Altogether the data show that partial inhibition of cellular respiration and subsequent ATP depletion up-regulated the cardiac lipid metabolism and induced signs of heart failure in 5 month-old ApoE^-/-^ mice.

### Myocardial Lipid Accumulation and Key Fat Synthesizing Enzymes in Cardiac Biopsy Specimens of Patients with Heart Failure

**Fig. (8):**
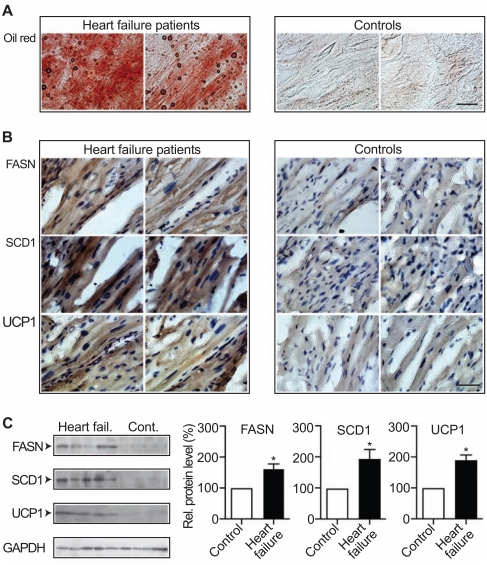
Myocardial lipid accumulation and lipid metabolizing enzymes in cardiac biopsy specimens of patients with heart failure. **A**. Oil red O (Oil red) staining of frozen heart sections showed cardiac lipid load in myocardial biopsy specimens isolated from patients with heart failure (Heart failure patients, n=2; left panels) compared to control patients (Controls, n=2; right panels), bar: 40 µm. **B**. Upper panels: immunohistological detection of FASN with F(ab)_2_ fragments of FASN-specific antibodies was performed in myocardial biopsy specimens of two patients with heart failure (left panels; Heart failure patients) relative to heart muscle specimens of two control patients (right panels; Controls). Middle panels: immunohistochemistry localized the key fatty acid synthesizing enzyme SCD1 in myocardial biopsy specimens of two patients with heart failure (left panels) whereas SCD1 was barely detectable with SCD1-specific antibodies in heart muscle biopsy specimens of two control patients (right panels). Lower panels show detection of high UCP1 protein levels in myocardial biopsy specimens of failing hearts (left panels; n=2) while UCP1 was almost undetectable in heart biopsies of control patients (right panels; n=2). Cell nuclei were stained with hematoxylin (bar: 40 µm). **C**. Left panels: Immunoblot detection of FASN, SCD1 and UCP1 in heart biopsy specimens of five patients with heart failure (Heart fail.) relative to four control patients (Cont.). As loading control, GAPDH was used. Right panels show quantifications of relative protein levels (expressed as percentage of control; %) of FASN, SCD1 and UCP1 in myocardial biopsy specimens of patients with heart failure (Heart failure; n=5) relative to control patients (Control; n=4) as determined by densitometric immunoblot blot scanning (± S.E.M.; *, p<0.05).

We next analyzed whether up-regulation of the cardiac fat metabolism was also a characteristic feature of human heart failure. Biopsy specimens of heart muscle were obtained from heart failure patients and from control patients without symptoms of heart failure during surgery for mitral valve replacement (Fig. **8**). Cardiac tissue from failing hearts was characterized by a high accumulation of lipids as detected by oil red O staining (Fig. **8A**, left panels). For comparison, cardiac biopsy specimens from patients without heart failure symptoms did not show significant oil red O staining (Fig. **8A**, right panels). 

Immunohistological analysis detected high protein levels of key fat synthesizing enzymes, FASN and SCD1, in the hypertrophic myocardial tissue of patients with heart failure (Fig. **8B**, left, upper and middle panels). In contrast, control specimens from patients without heart failure did not show significant levels of FASN and SCD1 (Fig. **8B**, right, upper and middle panels). Heart muscle specimens from patients with heart failure also showed significant levels of UCP1 protein relative to control specimens from patients without heart failure (Fig. **8B**, lower panels).

Immunoblot detection was used to quantify protein levels of FASN, SCD1 and UCP1 in cardiac tissue specimens from patients with heart failure. Quantitative immunoblotting confirmed the immunohistology data showing significantly increased protein levels of key lipid synthesizing and oxidizing enzymes FASN, SCD1 and UCP1 in cardiac tissue specimens from patients with symptoms of heart failure compared to cardiac biopsy specimens from control patients without heart failure (Fig. **8C**). Thus, lipid overload and up-regulation of the cardiac lipid metabolism were also present in heart specimens of patients with heart failure.

## DISCUSSION

### Identification of a Cardiac Lipid Metabolism Gene Cluster in Failing Hearts

Lipid overload in ischemic and failing heart muscle is established since decades [25, 33]. While the accumulation of fatty acids in ischemic and failing heart muscle is well known, underlying pathomechanisms are incompletely (± S.E.M.; *, p<0.05).

understood. The heart mainly relies on the uptake of fatty acids from the blood for energy generation, whereas the role of *de novo* synthesis of fatty acids by cardiac cells is negligible under normal conditions [25, 34]. In contrast to normoxic conditions, several studies have found that the capacity of cardiac cells of synthesizing fatty acids was increased under hypoxic conditions [35, 36]. However, data on the cardiac fat metabolism at the onset of heart failure (characterized by insufficient oxygen supply) are lacking. In view of the importance of lipids for cardiac energy generation and the reduced myocardial energy efficiency in heart failure, we analyzed the expression of fat metabolism genes at the onset of heart failure. Microarray gene expression and immunohistological analyses detected the strong up-regulation of a cluster of genes involved in fat synthesis, storage and oxidation in the failing myocardium of three different models of pressure overload- and severe atherosclerosis-induced heart failure. Moreover, up-regulation of key fat metabolizing enzymes was also present in cardiac biopsy specimens of patients with heart failure.

### Lipids Enhance Cardiomyocyte Death *In Vitro* and *In Vivo*

The up-regulation of the lipid metabolism in the failing heart correlated with an increased accumulation of cardiac lipids in different animal models and patients. Accumulation of lipids is detrimental to the myocardium as evidenced with isolated cardiomyocytes or genetic models of lipotoxic cardiomyopathy [27, 37-39]. Several possible mechanisms of injury could account for the deleterious effects of free fatty acids to the myocardium: (i) accumulation of toxic intermediates of FFA metabolism, (ii) inhibition of glucose utilization, and (iii) uncoupling of oxidative metabolism [40]. Our data are complementary to those studies showing that over-expression of the key palmitate synthesizing enzyme, Fasn, increased markers of cardiomyocyte death similarly as did palmitate. In agreement with lipotoxicity, up-regulation of the cardiac fat metabolism *in vivo* in different models of heart failure correlated with an enhanced rate of cardiomyocyte death during the transition from cardiac hypertrophy to overt cardiac failure.

### Catecholamines and Angiotensin II are Related to Cardiac Lipid Overload

Which factors trigger the cardiac fat metabolism during the development of heart failure? Heart failure is characterized by a maladaptive neurohormonal activation and catecholamine accumulation. It is well established that catecholamines exert major metabolic effects, which could finally culminate in enhanced lipogenesis and lipid overload: Catecholamines induce inappropriate FFA use by triggering (i) an increase in serum levels of FFAs, (ii) wasteful cycling of FFAs through enhanced intramyocardial lipolysis, and (iii) FFA-mediated suppression of glucose metabolism [41]. Concomitantly, catecholamines increase the RNA stability of FASN [42], and trigger UCP1 gene expression via β-adrenergic receptor-mediated stimulation of cAMP [43]. Apart from over-activation of the adrenergic system, the angiotensin II AT1 receptor, another key player in the development of heart failure, also up-regulates the cardiac fat metabolism by increasing the expression of FASN [44, 45]. The increased cardiac lipids could further stimulate the cardiac lipid metabolism by activation of transcriptional activators such as CCAAT/enhancer-binding protein (C/EBP) alpha (this microarray study, GSE25765-8, and ref. 44), and C/EBP alpha-dependent fat metabolism genes [46, 47]. Thus, neurohormonal activation during the pathogenesis of heart failure could be causally involved in enhancing the expression of fat metabolism genes and triggering lipid-induced lipid overload.

### Anti-Ischemic Treatment with Ranolazine Prevents Up-Regulation of the Cardiac Fat Metabolism and Improves Heart Failure Signs

As a direct or indirect consequence of neurohormonal activation, the failing heart suffers from increased oxygen and ATP consumption [3]. However, due to uncoupled and wasteful energy generation, oxygen supply is not sufficient to meet the increased ATP demand. In fact, cardiac ATP levels are decreased during the pathogenesis of heart failure [3]. Our data are complementary to those findings and indicate a causal relationship between insufficient oxygen and ATP supply, up-regulation of fat metabolism genes and heart failure because anti-ischemic treatment with ranolazine prevented the up-regulation of fat metabolism genes and improved heart failure signs in two different models of heart failure. Notably, ranolazine exerted its anti-ischemic effects without lowering blood pressure [29] or reducing atherosclerotic plaques. 

### Insufficient Oxygen and ATP Supply Trigger the Cardiac Fat Metabolism

A causal role of oxygen deprivation in the up-regulation of the cardiac fat metabolism is suggested by a previous finding, which shows that hypoxia could trigger the expression of key fatty acid synthesizing enzymes such as Scd1 [48]. Inhibition of cellular respiration and ATP generation by the mild thiol-blocking agent cystamine further supported the impact of oxygen/ATP deprivation for the cardiac lipid metabolism. Similarly as did pressure overload (imposed by aortic constriction), depletion of myocardial ATP content by cystamine not only up-regulated the cardiac lipid metabolism but also induced heart failure in ApoE^-/-^ mice within four weeks. Those experiments strongly indicate that ATP depletion, which is a characteristic feature of heart failure in patients and experimental models [2, 3, 49], is causally involved in triggering dysfunction of the cardiac lipid metabolism and toxic lipid overload. In agreement with that conclusion, heart biopsy specimens of patients with heart failure showed massive lipid accumulation and high protein levels of key fat metabolizing enzymes FASN, SCD1 and UCP1.

Altogether, the herein identified cluster of lipid metabolism genes could be directly triggered by chronic ATP depletion, which is a hallmark of heart failure [2, 3]. The resulting dysfunction of the essential cardiac lipid metabolism could initiate a vicious circle of lipid-induced fat synthesis. The rich supply of cardiac lipids cannot be used for ATP generation because accumulated lipids in concert with sympathetic over-stimulation induce the excessive accumulation of UCP, which uncouples fat oxidation from ATP generation [7, 43]. As a consequence toxic lipid overload develops and triggers cardiomyocyte death. Thus, despite of excessive substrate supply, cardiac energy depletion may occur, marking the transition from adaptive cardiac hypertrophy to heart failure. 

## CONCLUSION

The current study demonstrates that up-regulation of the cardiac lipid metabolism and toxic lipid overload mark the onset of heart failure in different experimental models of heart failure and patients. Triggered by oxygen/ATP depletion, cardiac lipid overload may promote heart failure by enhancing cardiomyocyte death. As a result of the study, a new treatment option of heart failure is proposed: Increased cardiac oxygen delivery by anti-ischemic treatment with ranolazine could normalize the cardiac lipid overload and improve signs of heart failure, at least in experimental models.

## ABBREVIATIONS

AAC: = Abdominal aortic constriction
ApoE: = Apolipoprotein E
ApoE^-/-^: = Apolipoprotein E-deficient
B6 mice: = C57BL/6J mice
Bdh: = 1, 3-hydroxybutyrate dehydrogenase type 1
C/EBP alpha: = CCAAT/enhancer-binding protein alpha
Fasn: = Fatty acid synthase
FFA: = Free fatty acid
Gapdh: = Glyceraldehyde-3-phosphate dehydrogenase
HE: = Hematoxylin and eosin
Nppa: = Natriuretic peptide precursor type A
Nppb: = Natriuretic peptide precursor type B
Retn: = Resistin
Scd1: = Stearoyl Coenzyme-A desaturase 1
Ucp: = Uncoupling protein
